# Plasma Prokineticin 1, a prognostic biomarker in colorectal cancer patients with curative resection: a retrospective cohort study

**DOI:** 10.1186/s12957-021-02421-0

**Published:** 2021-10-18

**Authors:** Noriyuki Tagai, Takanori Goi, Michiaki Shimada, Hidetaka Kurebayashi

**Affiliations:** Department of Surgery 1, University of Fukui, 23-3 Matsuokashimoaizuki, Eiheiji-Cho, Yoshida-gun, Fukui, 910-1193 Japan

**Keywords:** Colon cancer, Rectal cancer, Prokineticin 1, Prognostic biomarker, Plasma

## Abstract

**Background:**

Prokineticin 1 (PROK1) was reported as an angiogenic factor, which is associated with tumor progression, cell invasion, and metastasis in colorectal cancer. Although the association between PROK1 expression in primary cancer lesion and patient prognosis was reported, it is unclear whether plasma PROK1 concentration may be a predictive factor in colorectal cancer patients. This study investigated the association between PROK1 concentration in plasma and prognosis in colorectal cancer patients.

**Methods:**

We measured preoperative PROK1 plasma levels using ELISA method, while PROK1 expression in primary cancer lesion was evaluated using immunohistochemistry (IHC). The association between plasma PROK1 levels and cancer-related survival rate (CRS) was evaluated. Additionally, we examined whether simultaneous PROK1 expression in both primary cancer lesions and plasma was correlated with CRS. The cancer-related survival rate was calculated using the Kaplan-Meier method, and survival estimates were compared using the log-rank test.

**Results:**

We have gathered eligible 130 CRC patients retrospectively. Out of 130 patients, 61 (46.9%) were positive on IHC in primary cancer, and 69 (53.1%) were negative, while 43 (33.1%) had high-value PROK1 in plasma. Out of these 43, 30 (25.4%) also had concomitant higher IHC expression in primary cancer. The plasma PROK1 levels tended to increase with advancing stages. The plasma PROK1-positive group had a lower 5-year CRS than the negative group (63.6% vs. 88.2%; *P* = 0.006). Additionally, simultaneous PROK1 expression was associated with a more significant decrease of 5-year CRS than both negative groups in all stages (76.2% vs. 92.5%; *P* = 0.003) and stage III (59.3% vs. 84.5%; *P* = 0.047). Multivariate analysis showed simultaneous PROK1 expression was independently associated with worse CRS (HR, 1.97; 95% CI 1.20‑3.24, *P* < 0.01).

**Conclusion:**

PROK1 expression in preoperative plasma reflects poor prognosis in patients undergoing curative resection for colorectal cancer. The plasma PROK1 level may be a potential predictive marker, especially in stage III colorectal cancer patients.

**Supplementary Information:**

The online version contains supplementary material available at 10.1186/s12957-021-02421-0.

## Background

Colon cancer is the third most common cancer in the world. Further improvement of the treatment for that cancer still has been required [1]. Some guidelines in the world recommend adjuvant chemotherapy for curatively resected stage III colon cancer [2–4]. Although the benefit of the adjuvant chemotherapy was shown as an approximately 20% reduction in the risk of recurrence and mortality [5], a certain number of patients can be cured without adjuvant chemotherapy. On the other hand, neurotoxicity remains a major problem in the use of oxaliplatin-based regimens for stage III colorectal cancer patients. The adverse event sometimes becomes persistent and interferes with daily activity [6]. From these aspects, we should select high-risk patients who really require adjuvant chemotherapy and avoid unnecessary application of adjuvant chemotherapy. However, no useful biomarker has been applied in a clinical setting, although various predictive biomarker candidates have been evaluated in recent years [7].

Angiogenic growth factors have been considered to play an important role in the proliferation of colorectal cancer [8]. Some drugs targeting vascular endothelial growth factor (VEGF) have been applied in clinical practice for advanced colorectal cancer [9]. Endocrine gland-derived vascular endothelial growth factor (EG-VEGF/Prokineticin 1[PROK1]) is an angiogenic factor mainly present in the endocrine glands [10]. PROK1 is a member of the prokineticin family, while another member is prokineticin 2 (PROK2), which is also reported as an angiogenic factor [11]. PROK1 and PROK2 activate two G protein-coupled receptors: Prokineticin receptor 1 (PKR1) and Prokineticin receptor 2 (PKR2). Stimulated these receptors activate the mitogen-activated protein kinase (MAPK) pathway and the phosphatidylinositol 3 kinase (PI3K)/Akt pathway leading to cell proliferation, angiogenesis [12, 13]. Additionally, PROK1 may activate tumor invasion via matrix metalloproteinases (MMP), especially MMP2, MMP7, and MMP9 [14].

Recent studies reported that PROK1 expressed not only endocrine glands but also some malignant tumors, including prostate cancer [15], pancreatic cancer [16], ovarian cancer [17], adrenocortical tumors [18], and colorectal cancer [19]. In colon cancer, it has been reported that PROK1 expression in primary cancer lesions was associated with tumor recurrence and patient prognosis [20]. However, it is unclear whether the concentration of PROK1 in the blood of patients can be used as a new prognostic biomarker for patients with colorectal cancer. The preoperative selection of high-risk patients may lead to the indication of neoadjuvant chemotherapy.

The present study aimed to examine the association between preoperative PROK1 concentration in the plasma of patients with colorectal cancer and their prognosis.

## Methods

### Patients and samples

This was a single-center, cohort study. Eligible subjects were chemotherapy-naive colorectal cancer patients who underwent surgery with D2 or D3 lymph node dissection (stages I‑IV: UICC-TNM 8th [21]) and underwent R0 resection at the Department of Surgery 1, University of Fukui, Japan, between 2011 and 2016. Two pathologists in the pathological department at the University of Fukui Hospital evaluated pathological findings of surgical specimens. Patients excluded were as follows: followed up less than 3 years after surgery, did not undergo D2 or D3 lymph node dissection, had multiple primary cancers, were diagnosed with a primary cancer lesion no deeper than the lamina propria (pTis), and without R0 resection. Twenty milliliters of blood samples was collected before surgery from each patient in the morning on the operation day following the addition of 1000 U unfractionated heparin (50 U/mL). Plasma was collected after centrifugation at 800 g for 10 min, and the samples were frozen at −80 °C. Surgical specimens, including primary cancer tissues, were fixed in 10% paraformaldehyde (pH 6.8) for 24 h and embedded in paraffin.

### Measurement of plasma concentration of PROK1 using ELISA

The concentration of PROK1 in plasma was measured using an enzyme-linked immunosorbent assay (ELISA) with the OmniKine (Human) EG-VEGF ELISA kit (Assay bio-Tech, California, USA) according to the manufacturer’s instructions. The test kit showed less than 1% cross-reactivity with human Prokineticin-2 by the manufacturer. The cut-off points for PROK1 concentration were determined using the receiver operating characteristic (ROC) curve. We determined the concentration above the cut-off value as positive and below the value as negative.

### Immunohistochemical (IHC) study

Paraffin sections were cut into 4-μm sections, deparaffinized with xylene, and dehydrated through a graded ethanol series. Deparaffinized sections were incubated with 1% hydrogen peroxidase in methanol for 30 min to reduce endogenous reactivity. After high-temperature antigen retrieval in an autoclave at 121 °C for 15 min, non-specific reactivity was blocked by a dilution of skim milk powder for 30 min. These sections were incubated overnight at 4 °C with the rabbit anti-human PROK1 polyclonal antibody (NOVUS Biochemicals, Colorado, USA) diluted 1:100 using antibody diluent, Dako REAL^TM^ (DAKO, Agilent, CA, USA). After three washes with phosphate-buffered saline (PBS), we used the Envision system (DAKO, Agilent, California, USA) for the application of a secondary antibody for 30 min at room temperature and washed out the solution with Tris-buffered saline (TBS). Diaminobenzidine was applied to the sections for 4 min to evaluate the expression of PROK1, and then immersed in a hematoxylin solution for counterstaining. We picked up 5 hot spots in cancer tissue and measured the area of cancer tissue using the Image J software. We categorized staining intensity into three grades: weak, moderate, and strong. While we considered moderate and strong staining areas as positive, we calculated the average of the percentage in PROK1 positive area among five hot spots. We then determined it positive if the average was more than 30% [20].

### Statistical analysis

The correlation between PROK1 expression and clinicopathological findings was statistically assessed using Fisher’s exact test. The association between the concentration of PROK1 in the plasma and the pathological stages (UICC-TNM 8th edition) was evaluated using the Kruskal-Wallis test. Receiver operating characteristic (ROC) curves for cancer-related survival (CRS) were used to determine the optimal cut-off point for the concentration of PROK1. The association between PROK1 expression in the plasma and primary cancer tissues was also evaluated using the chi-square test. The cancer-related survival rate was calculated using the Kaplan-Meier method, and survival estimates were compared using the log-rank test. We used Cox proportional hazards regression model to estimate hazard ratio. Univariate and multivariate analyses were also conducted using Cox regression model. All statistical analyses were performed with EZR (ver.1.37) (Saitama Medical Center, Jichi Medical University, Saitama, Japan), which is a graphical user interface for R (The R foundation for Statistical Computing, Vienna, Austria). More precisely, it is a modified version of R commander designed to add statistical functions frequently used in biostatistics [22]. All *P* values of 0.05 or less were considered statistically significant.

## Results

**Fig. 1:**
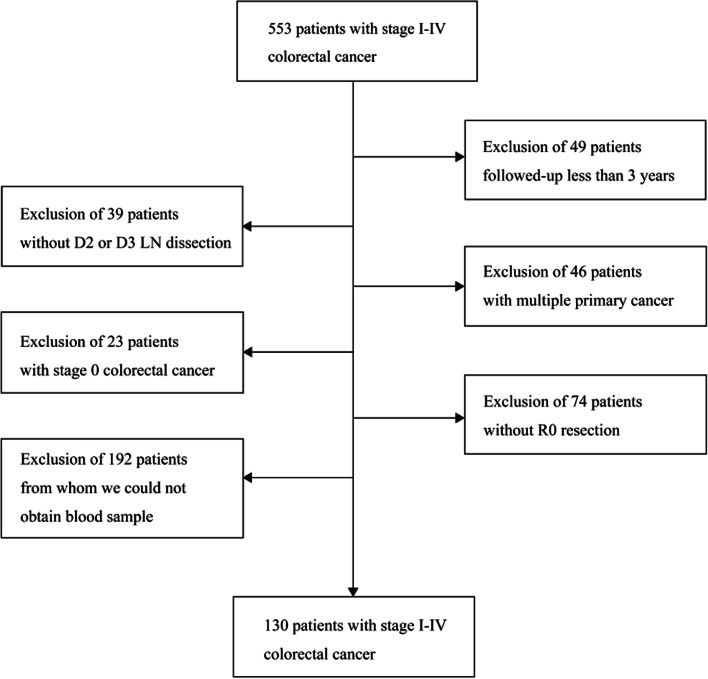
Process flow outlining patient enrollment

In this study, 553 patients were assessed for eligibility. Excluded patients were as follows: 49 patients for short follow-up duration, 39 patients for the lack of enough lymph-node dissection, 46 patients for multiple primary cancer, 23 patients for pTis in the depth of a primary cancer lesion, and 74 patients for R1 or R2 resection. Additionally, blood sample data from 192 patients were not recovered in our institution and were excluded from this study (Fig. 1). All of the eligible patients were treated based on Japanese Guidelines for the Treatment of Colorectal Cancer.

**Fig. 2:**
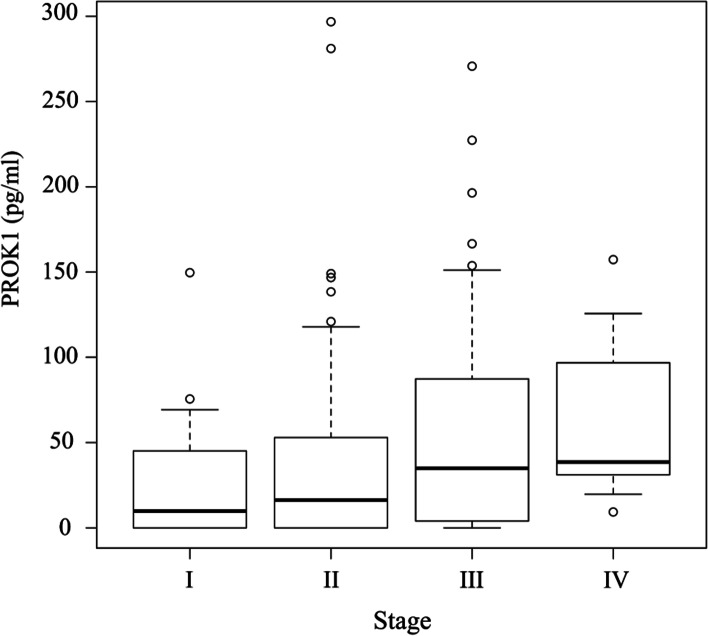
A box-plot analysis in the concentration of plasma PROK1 in each stage. A box-plot analysis showed that the concentration of PROK1 tended to increase with the advancement of the stage, although the difference was not significant (*P* = 0.31)

**Fig. 3:**
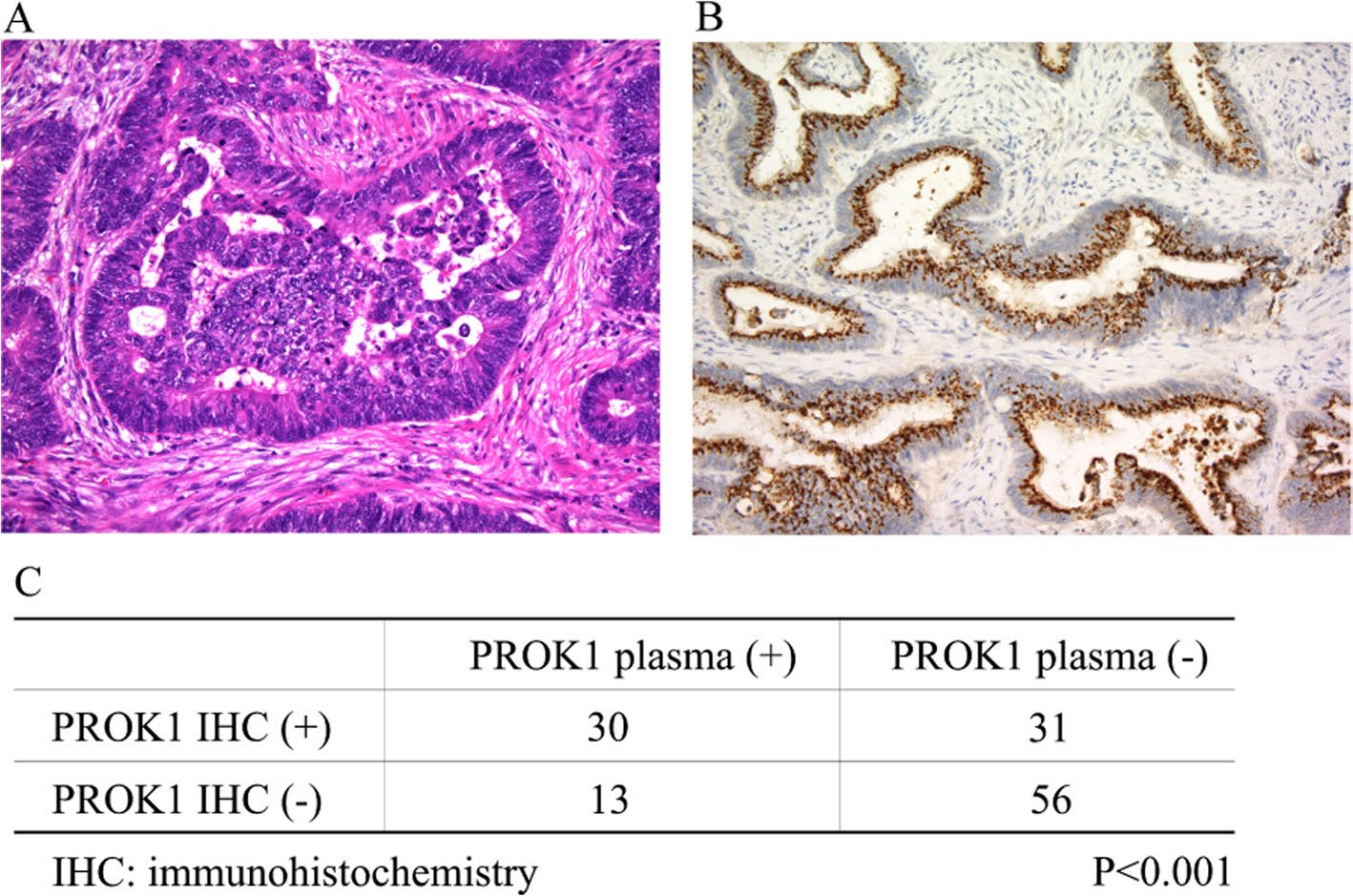
The association between plasma PROK1 and PROK1 expression in primary cancer lesion. **A** A representative picture of the specimen stained with hematoxylin-eosin (magnification, × 200). **B** A representative picture of PROK1-positive primary cancer lesion (magnification, × 200). The PROK1 was positive in the cytoplasm of cancer cells. In this case, most of the cancer cells were positive for PROK1. **C** The plasma PROK1-positive patients were identified more commonly in the immunohistochemistry (IHC) positive group than the IHC negative group

In total, 130 patients were enrolled, and their plasma PROK1 concentration was measured. The cut-off level of PROK1 for CRS was 56.9 pg/mL (area under the curve (AUC), 0.62; negative likelihood ratio, 0.56; positive likelihood ratio, 1.48; and diagnostic odds ratio, 2.45; sensitivity, 70.6%; specificity, 50.2%) using the ROC curve (Additional file 1). The concentration of PROK1 among patients with stages I‑IV is described in Fig. 2. Although PROK1 concentration tended to increase with the advancement of stages, the difference was not statistically significant (*P* = 0.31). Immunohistochemistry (IHC) revealed that 61 (46.9%) patients were positive for PROK1 expression in the primary tumor, and 30 (25.4%) of them were positive for PROK1 in the plasma (Fig. 3). PROK1 in the primary cancer lesion positive group included more plasma PROK1-positive patients than in the negative group (*P* < 0.001). Patient characteristics are shown in Table 1. In this cohort, there was no significant difference among the factors in the table.

**Table 1:** Patients’ characteristics

	Plasma PROK1 positive (%)	Plasma PROK1 negative (%)	*P* value
All cases	43 (33.1)	87 (66.9)	
Gender			0.27
Male	20 (28.6)	50 (71.4)	
Female	23 (38.3)	37 (61.7)	
Age			0.57
< 65	20 (36.4)	35(63.6)	
≧ 65	23 (30.7)	52 (69.3)	
Location			0.44
Right colon	17 (38.6)	27 (61.4)	
Left colon	26 (30.2)	60 (69.8)	
Histological type			0.5
Well+mod+pap	38 (31.9)	81 (68.1)	
Por or muc	5 (45.5)	6 (54.5)	
Lymphatic invasion			0.1
Negative	1 (8.3)	11 (91.7)	
Positive	42 (35.6)	76 (64.4)	
Venous invasion			1
Negative	8 (30.8)	18 (69.2)	
Positive	35 (33.7)	69 (66.3)	
Peritoneal metastasis			1
Negative	43 (33.3)	86 (66.7)	
Positive	0 (0)	1 (100)	
Hematogenous metastasis			0.6
Negative	41 (32.5)	85 (67.5)	
Positive	2(50)	2(50)	
T (UICC-TNM 8th)			0.89
T1	3 (27.3)	8 (72.7)	
T2	6(30)	14(70)	
T3	17 (31.5)	37 (68.5)	
T4	17 (37.8)	28 (62.2)	
N (UICC-TNM 8th)			0.078
N0	22 (27.5)	58 (72.5)	
N1	20 (40.8)	29 (59.2)	
N2	1 (100)	0 (0)	
Stage (UICC-TNM 8th)			0.2
I	4 (20)	16 (80)	
II	16 (28.1)	41 (71.9)	
III	20 (43.5)	26 (56.5)	
IV	3 (42.9)	4 (57.1)	
Adjuvant chemotherapy (each stage)			0.12
I	0 (0)	0 (0)	
II	4 (16.7)	20 (83.4)	
III	15 (40.5)	22 (59.5)	
IV	1 (20)	4 (80)	

*PROK1* Prokineticin 1, *UICC* The Union for International Cancer Control, *Well* well-differentiated carcinoma, *mod* moderately differentiated carcinoma, *pap* papillary carcinoma, *Por* poorly differentiated carcinoma, *muc* mucinous carcinoma

### Plasma PROK1 expressions in colorectal cancer patients and their prognosis

**Fig. 4:**
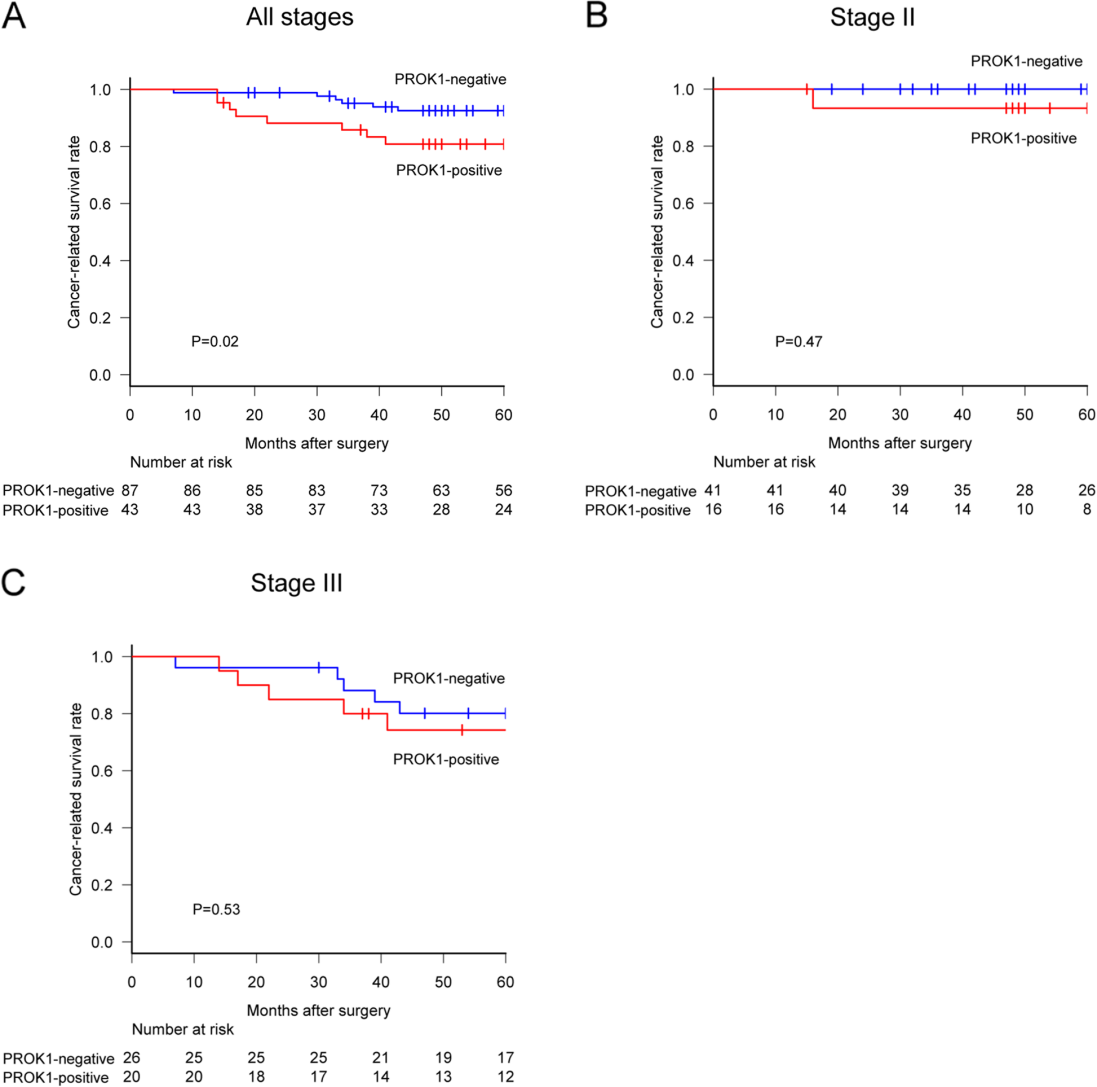
Plasma PROK1 expression affected the prognosis of patients. **A** The Plasma PROK1-positive group showed a poorer prognosis than that of the negative group in all stages. **B** In stage II patients, there was no significant difference between the two groups. **C** In stage III patients, there was no significant difference between the two groups

**Fig. 5:**
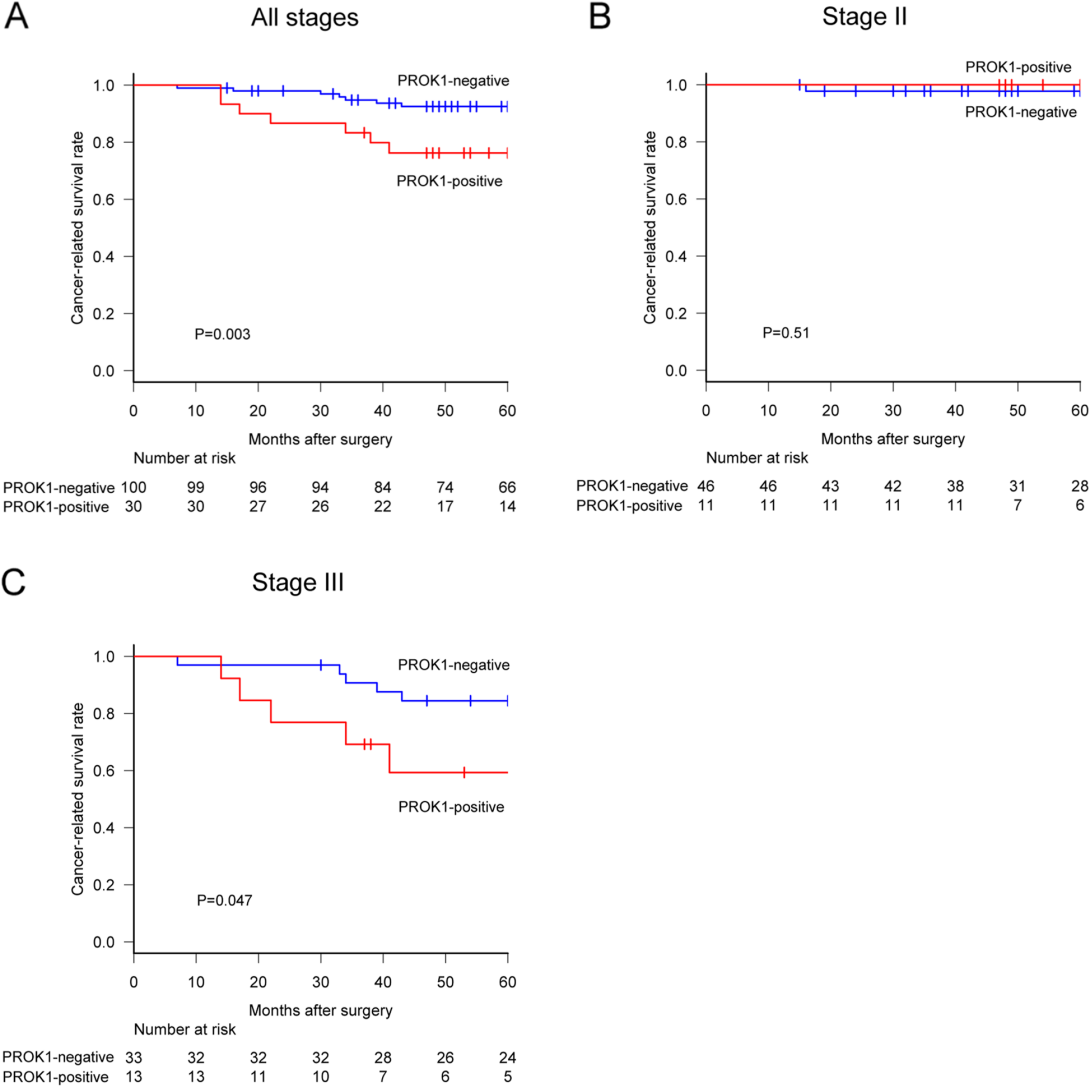
The simultaneous PROK1 expression in both plasma and primary cancer lesion strongly affected patient prognosis. **A** The PROK1-positive group showed poorer CRS than that of the negative group in all stages. **B** In stage II patients, there was no significant difference. **C** In stage III patients, the PROK1-positive group was strongly associated with a lower CRS than that of the negative patients

The median follow-up duration of the subjects was 62.5 months. Among all patients, the 5-year cancer-related survival rate in the plasma PROK1-positive group was significantly lower than that in the negative group (80.8% [95% CI 84.2‑96.6] vs. 92.6% [95% CI 65.3‑89.9], HR 2.68 [95% CI 1.13‑6.33], *P* = 0.02, Fig. 4A). The 5-year cancer-related survival rates in patients with stage II disease were 93.3% (95% CI 61.3‑99) in the plasma PROK1-positive group and 100% (95% CI 100‑100) in the plasma PROK1-negative group (HR 2.43 [95% CI 0.21‑28.5], *P* = 0.47, Fig. 4B). Among the patients with stage III disease, the rates were 74.3% (95% CI 48.7‑88.4) in the positive group and 80.1% (95% CI 58.6‑91.2) in the negative group (HR 1.4, [95% CI 0.49‑3.99], *P* = 0.53, Fig. 4C). Considering the association between PROK1 in plasma and primary cancer lesions, the 5-year cancer-related survival rate of the positive group for PROK1 in both plasma and primary cancer lesions was 76.2% (95% CI 56.5‑87.9) and 92.5% (95% CI 84.9‑96.4) in both the negative group (HR 1.9 [95% CI 1.21‑2.96], *P* = 0.003) (Fig. 5A). The 5-year cancer-related survival rate with stage II disease was 100% (95% CI 100‑100) in both the plasma and IHC positive group and 97.8% (95% CI 85.3‑99.7) in both the plasma and IHC negative groups (*P* = 0.51, Fig. 5B). Among the patients with stage III disease, the rates were 59.3% (95% CI 27.5‑81) in both positive group and 84.5% (95% CI 66.6‑93.2) in both negative group (HR 1.69 [95% CI 0.98‑2.89], *P* = 0.047, Fig. 5C). PROK1 positive group showed relatively higher recurrence rate, although the difference was not statistically significant. Disease-free survival rate was lower in PROK1 positive group than that of negative group (*P* = 0.012, Table 2).

**Table 2:** The correlation between PROK1 expression and cancer recurrence

	Plasma and IHC PROK1 (+)	Plasma and IHC PROK1 (−)	*P* value
All cases	30	100	
Recurrence (%)	11 (36.7)	20 (20)	0.1
Liver	6	8	
Lung	2	6	
Para-aortic LN	0	3	
Peritoneal	2	1	
Others	2	6	
5-year DFS	52.3% (95% CI 32.9‑68.8)	73.4% (95% CI 63.5‑81.1)	0.012

*IHC* immunohistochemistry, *LN* lymph node, *DFS* disease-free survival

PROK1 positive group showed relatively higher recurrence rate, although the difference was not statistically significant. Disease-free survival rate was lower in PROK1 positive group than that of negative group (*P* = 0.012). The number of patients in each recurrence site were overlapped due to some patients relapsed with more than one metastatic site.

Univariate analysis for CRS showed that the simultaneous PROK1 expression and stage are significant prognostic factors. Besides, the simultaneous PROK1 expression was also independently associated with worse CRS (HR, 1.97; 95% CI 1.20‑3.24; *P* < 0.01, Table 3).

**Table 3:** Univariate and multivariate analysis for cancer-related survival

	Univariate analysis		Multivariate analysis	
	HR (95% CI)	*P* value	HR (95% CI)	*P* value
Age over 65	0.90 (0.38‑2.13)	0.82		
Sex (male vs. female)	1.18 (0.50‑2.80)	0.71		
Simultaneous PROK1 expression	1.90 (1.21‑2.96)	< 0.01	1.97 (1.20‑3.24)	< 0.01
Stage	3.93 (2.11‑7.32)	< 0.01	4.36 (2.26‑8.44)	< 0.01

*HR* hazard ratio, *CI* confidence interval

### The association between PROK1 expression and the prognosis of patients treated with adjuvant chemotherapy

**Fig. 6:**
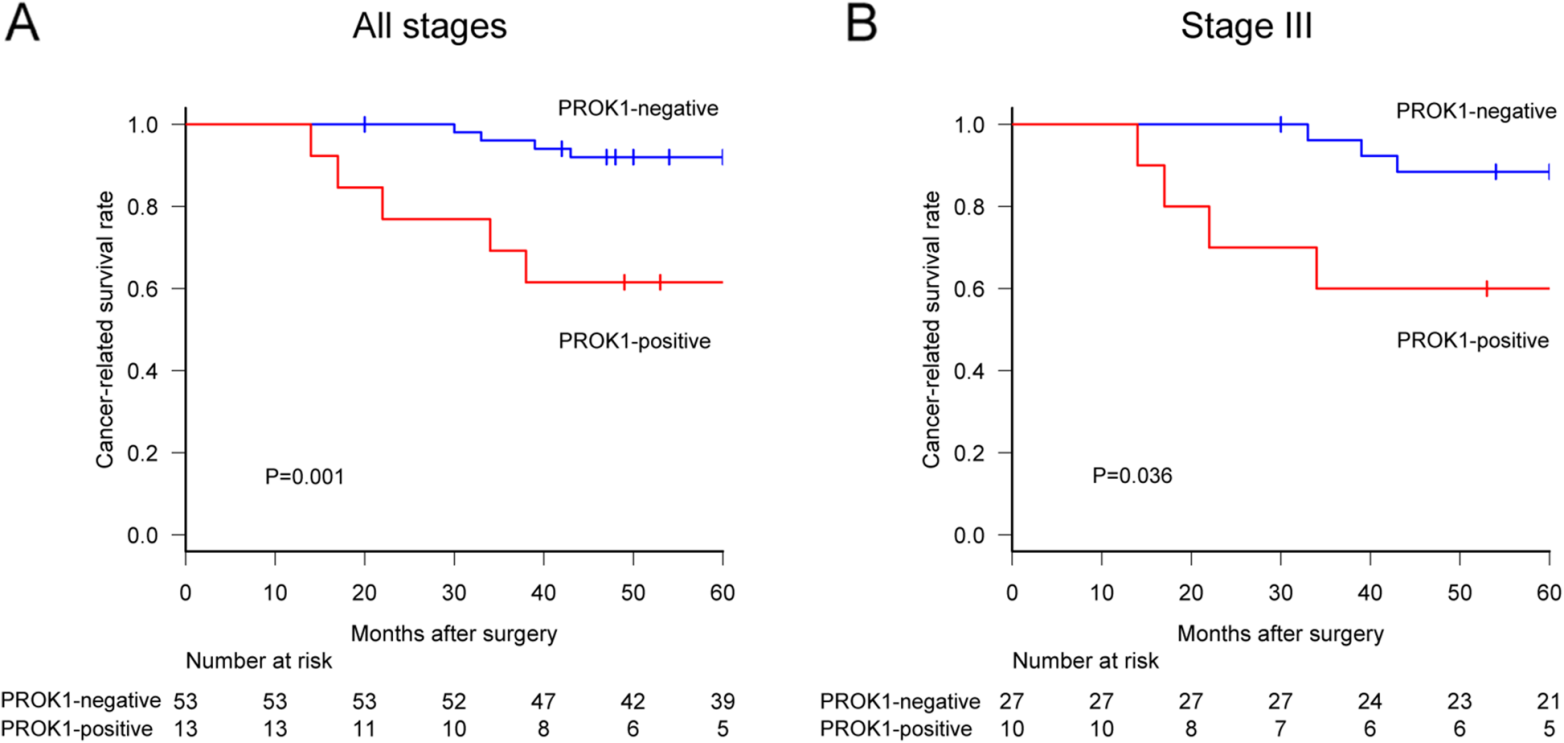
The simultaneous PROK1 expression in plasma and primary cancer lesion was associated with a lower CRS even among people who received adjuvant chemotherapy. **A** In all stages, the PROK1-positive group showed a lower CRS than that of the negative group. **B** Among stage III patients, the PROK1-positive group was clearly associated with a lower CRS

Considering the association between adjuvant chemotherapy and PROK1 expression, even among the patients who received adjuvant chemotherapy, the PROK1-positive group showed a lower 5-year cancer-related survival rate than the negative group (61.5% [95% CI 30.8‑81.8] vs. 92% [95% CI 80.1‑96.9], HR 2.27 [95% CI 1.30‑3.94], *P* = 0.001, Fig. 6A). Among stage III patients receiving adjuvant chemotherapy, the PROK1-positive group showed poorer prognosis in 5-year CRS than the negative group (60% [95% CI 25.3‑82.7] vs. 88.5% [95% CI 68.4‑98.1], HR 1.83 [95% CI 1.00‑3.33], *P* = 0.036, Fig. 6B).

## Discussion

This study investigated the association between plasma PROK1 expression and prognosis in patients with colorectal cancer undergoing R0 resection. The plasma PROK1-positive patients showed a lower 5-year cancer-related survival rate than patients without it. In terms of PROK1 expression in both plasma and primary cancer lesions, the simultaneous PROK1 expression was associated with a poorer prognosis than that in patients without simultaneous PROK1 expression especially among stage III patients.

PROK1 was first reported to be an angiogenic factor expressed in endocrine glands [10]. PROK1 has been shown to exert promoting tumor progression, invasion, and metastasis in colorectal cancer [23]. The mRNA expression of PROK1 showed poor prognosis in colorectal cancer [19], while 36% of primary cancer lesions were positive for PROK1 in immunohistochemistry, leading to a poorer prognosis [20]. In terms of PROK1 concentration in the plasma, one study reported that PROK1 expression in the plasma was associated with peritoneal carcinomatosis in colorectal cancer [24]. In our study, we revealed the association between plasma PROK1 expression and cancer-related survival rate in colorectal cancer patients undergoing R0 resection. Furthermore, PROK1 expression in both plasma and primary cancer lesions was more strongly associated with a lower 5-year cancer-related survival rate, especially in stage III disease.

Our results showed the association between circulating PROK1 in peripheral blood and patient prognosis. This result might be related to the expression of the prokineticin receptor in metastatic organs and their functions. For instance, prokineticin receptor 2 (PKR2) expression was detected in human hepatic sinusoidal endothelial cells (HHSECs), and the stimulation of PKR2 induces the disorganization of tight junctions [25]. This mechanism might contribute to fenestration in HHSECs and subsequent hepatic metastasis. Moreover, PKR2 expression in primary colorectal cancer lesions is also associated with poor prognosis of patients [26]. PROK1 may increase cell invasion capacity via PKR2 with high expression of matrix metalloproteinase 2, 7, and 9, contributing to aggressive metastasis and poor prognosis [14]. These two mechanisms at primary cancer lesion and distant organs may help to explain the poor patient prognosis.

This study showed the possibility of using PROK1 as a high-risk marker for stage III colorectal cancer. This may contribute to the selection of high-risk patients who genuinely need adjuvant chemotherapy. On the other hand, even in patients who underwent adjuvant chemotherapy, PROK1 expression showed a poor prognosis. The high level of plasma PROK1 may help us determine to extend the duration of adjuvant chemotherapy. While preoperative circulating PROK1 may reflect the prognosis of patients, this value may contribute to deciding whether neoadjuvant chemotherapy should be administered. An ELISA method could give results in a quantitative form, contributing to more precise decision-making than IHC. Additionally, we were able to obtain blood samples less invasively, allowing repeated measurements before and after chemotherapy. The change in circulating PROK1 expression during chemotherapy can be a topic for further study.

Our study had several limitations. First, while it was a single-center, retrospective cohort study, the enrolled patients did not receive the same treatment. However, the patients in the cohort were treated based on Japanese Society for Cancer of the Colon and Rectum (JSCCR) guidelines for the treatment of colorectal cancer. In this context, they were treated with standard treatment strategy in Japan. Second, blood samples were extracted from a limited number of patients who expressed their agreement with this study. The blood samples were obtained with different durations. While plasma samples were stored at −80 °C, their status might have changed over time. Third, the pathway of PROK1 secretion to plasma is poorly documented, while we did not focus on that mechanism in this study. Fourth, a little discrepancy of PROK1 expression between plasma and primary cancer lesion was shown in our study. Although this point is future challenge, the difference may be due to the cut-off point of plasma PROK1. Additionally, PROK1 in plasma may come from both primary cancer lesion and other organs naturally producing PROK1. Fifth, we could not obtain information about RAS status because the measurement of RAS status was covered by Japanese insurance in 2015. Microsatellite instability was also covered by Japanese insurance in 2018. We did not measure LINE-methylation status in this study. Further studies are required to examine the cause of the increase in PROK1 expression, including the association between PROK1 and tumor microenvironment. Additionally, there is a need for prospective studies to test our findings in a larger population in the future.

In conclusion, PROK1 expression in the plasma was associated with poor prognosis in patients with colorectal cancer who underwent R0 resection. Although further prospective large-scale studies are needed to confirm our results, the findings may contribute to the selection of high-risk patients who genuinely need adjuvant chemotherapy, particularly in stage III patients.

## Supplementary Information

**Additional file 1.** ROC analysis of plasma PROK1 for cancer-related survival. The cut-off level of PROK1 for CRS was 56.9 pg/mL (area under the curve [AUC)]: 0.62, negative likelihood ratio: 0.56, positive likelihood ratio: 1.48, and diagnostic odds ratio: 2.45, sensitivity: 70.6%, specificity: 50.2%).

## Data Availability

The datasets used and analyzed during the current study are available from the corresponding author on reasonable request.

## Abbreviations

PROK1: Prokineticin 1
CRS: Cancer-related survival rate
EG-VEGF: Endocrine-derived vascular endothelial growth factor
ELISA: Enzyme-linked immunoassay
ROC: Receiver operating characteristic curve
IHC: Immunohistochemistry
HHSEC: Human hepatic sinusoidal epithelial cells
PKR: Prokineticin receptor
