# Supplementary figures and images for: Plasma Prokineticin 1, a prognostic biomarker in colorectal cancer patients with curative resection: a retrospective cohort study
Source: World J Surg Oncol. 2021 Oct 18;19:302. doi: 10.1186/s12957-021-02421-0 (PMC8522247; doi:10.1186/s12957-021-02421-0)

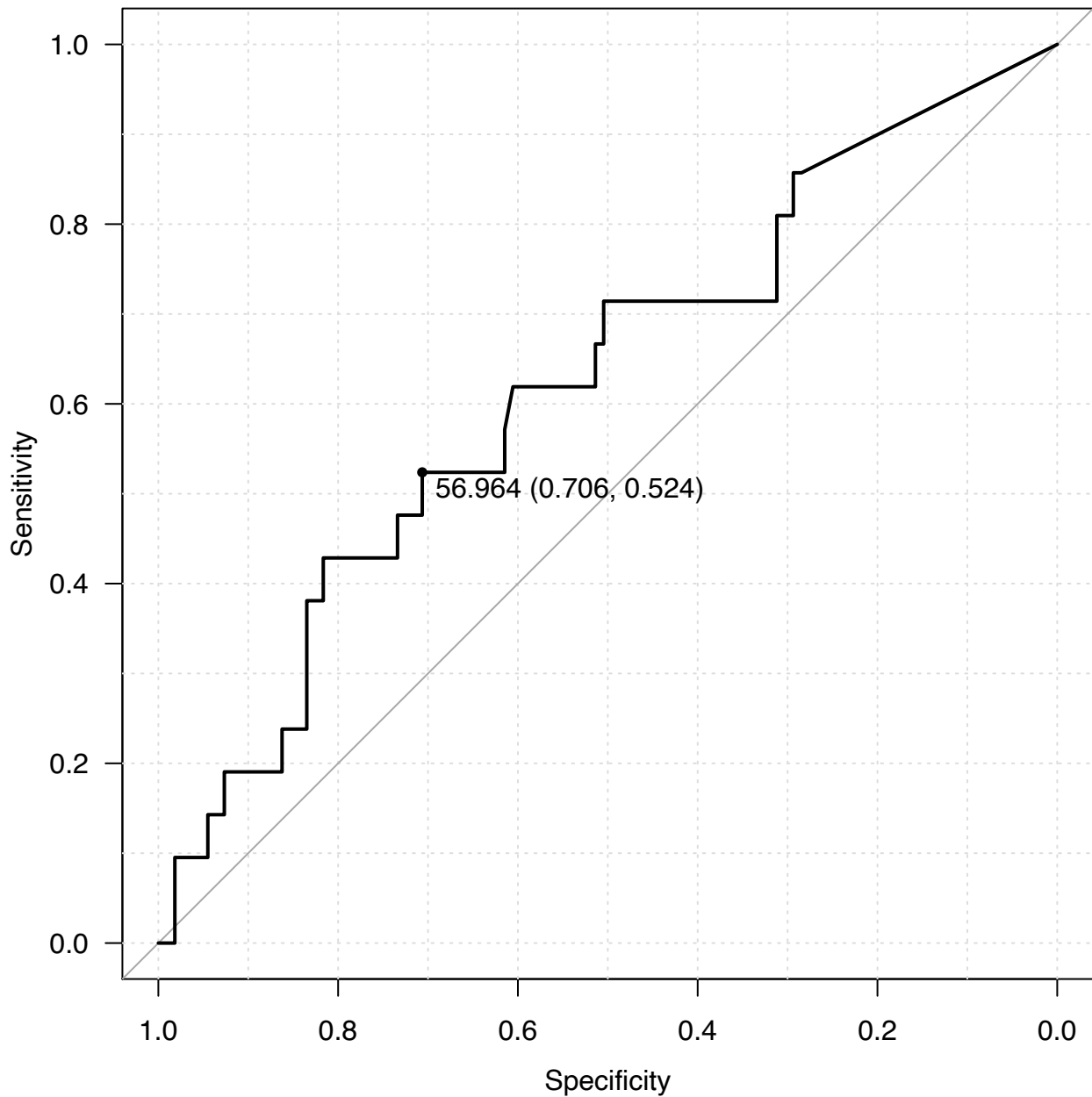

Supplement: Supplementary file 1 — Additional file 1. ROC analysis of plasma PROK1 for cancer-related survival. The cut-off level of PROK1 for CRS was 56.9 pg/mL (area under the curve [AUC)]: 0.62, negative likelihood ratio: 0.56, positive likelihood ratio: 1.48, and diagnostic odds ratio: 2.45, sensitivity: 70.6%, specificity: 50.2%). [file 12957_2021_2421_MOESM1_ESM.pdf]
